# Parecoxib reduced ventilation induced lung injury in acute respiratory distress syndrome

**DOI:** 10.1186/s40360-017-0131-z

**Published:** 2017-03-29

**Authors:** Fan-you Meng, Wei Gao, Ying-nan Ju

**Affiliations:** 1 Department of Anesthesiology, the Second Affiliated Hospital of the Harbin Medical University, Harbin, 150081 Heilongjiang Province China; 2 Department of Intensive Care Unit, the Third Affiliated Hospital of the Harbin Medical University, Harbin, 150081 Heilongjiang Province China

**Keywords:** Parecoxib, Ventilation induced lung injury, Acute respiratory distress syndrome

## Abstract

**Background:**

Cyclooxygenase-2 (COX-2) contributes to ventilation induced lung injury (VILI) and acute respiratory distress syndrome (ARDS). The objective of present study was to observe the therapeutic effect of parecoxib on VILI in ARDS.

**Methods:**

In this parallel controlled study performed at Harbin Medical University, China between January 2016 and March 2016, 24 rats were randomly allocated into sham group (S), volume ventilation group/ARDS (VA), parecoxib/volume ventilation group/ARDS (PVA). Rats in the S group only received anesthesia; rats in the VA and PVA group received intravenous injection of endotoxin to induce ARDS, and then received ventilation. Rats in the VA and PVA groups were treated with intravenous injection of saline or parecoxib. The ratio of arterial oxygen pressure to fractional inspired oxygen (PaO_2_/FiO_2_), the wet to dry weight ratio of lung tissue, inflammatory factors in serum and bronchoalveolar lavage fluid (BALF), and histopathologic analyses of lung tissue were examined. In addition, survival was calculated at 24 h after VILI.

**Results:**

Compared to the VA group, in the PVA group, PaO_2_/FiO_2_ was significantly increased; lung tissue wet to dry weight ratio; macrophage and neutrophil counts, total protein and neutrophil elastase levels in BALF; tumor necrosis factor-α, interleukin-1β, and prostaglandin E_2_ levels in BALF and serum; and myeloperoxidase (MPO) activity, malondialdehyde levels, and Bax and COX-2 protein levels in lung tissue were significantly decreased, while Bcl-2 protein levels were significantly increased. Lung histopathogical changes and apoptosis were reduced by parecpxib in the PVA group. Survival was increased in the PVA group.

**Conclusions:**

Parecoxib improves gas exchange and epithelial permeability, decreases edema, reduces local and systemic inflammation, ameliorates lung injury and apoptosis, and increases survival in a rat model of VILI.

## Background

Mechanical ventilation (MV) is an essential therapy for patients with severe lung injury or respiratory dysfunction. However, large volume and long-term ventilation can cause ventilation induced lung injury (VILI) [1]. MV with large tidal volume can result in regional overdistention of alveoli and airways, and the release of pro-inflammatory cytokines and prostanoids. This can cause local inflammation and lung injury [2], as well as systemic inflammation that can contribute to multiple organ failure and death.

Patients with acute respiratory distress syndrome (ARDS) or prior lung injury usually require MV support to guarantee oxygenation [3], and are particularly susceptible to VILI. Although lung protective strategies and other therapies are used during MV in ARDS patients, the therapeutic effect remains unsatisfactory [4–6]. Cyclooxygenase-2 (COX-2) plays an important role in inflammation, and inhibition of COX-2 production can attenuate local or systemic inflammation, including the production of chemokines and pro-inflammatory cytokines [7–9]. Evidence suggests that COX-2 inhibitors reduce pulmonary inflammation in animal models of lung injury [10–13], COX-2 contributes to ARDS [14, 15] and VILI [16], and inhibition of COX-2 reduces VILI [17] or acute lung injury induced by pancreatitis [7]. However, COX-2 inhibition or gene disruption is not currently applicable in the clinic. A previous report from our laboratory indicates that parecoxib, a COX-2–specific inhibitor that has widespread clinical use, attenuates lung injury induced by meconium in rabbits [13]. There are no published studies investigating the long term effect of parecoxib on VILI in ARDS. In the current study, we investigated the effect of parecoxib on VILI in a rat model of ARDS, considering short and long term outcomes. The findings may provide a novel treatment for ARDS patients undergoing MV.

## Methods

### Study design

Twenty-four adult (275-375 g) male Wistar rats were purchased from the Second Affiliated Hospital of Harbin Medical University, Harbin, China. The present study was approved by the Ethics Committee of Harbin Medical University, China. All experiments were performed in accordance with the guidelines of Institutional Animal Care and Use Committee of Harbin Medical University, China. Rats were fasted for 24 h before the study, but water was provided *ad libitum.*

Rats were randomly allocated into: sham group (S), large volume ventilation group/ARDS (VA), parecoxib/large volume ventilation group/ARDS (PVA). All rats were anesthetized with 3% pentobarbital sodium (30 mg/kg) and rocuronium 0.6 mg/kg by intraperitoneal injection. Rats in the VA and PVA groups were intubated and administered an intravenous injection of endotoxin 500 μg/kg (Escherichia coli endotoxin, 0111:B4, Sigma, Saint Louis, Missouri, USA). Then, rats in the VA and PVA groups were ventilated (tidal volume 20 ml/kg, respiratory rate: 50/min, FiO_2_: 50%, inspiratory:expiratory ratio: 1:1) for 4 h. Rats in the VA and PVA groups were administered an intravenous injection of saline or parecoxib 40 mg/kg (Pfizer, Kalamazoo, MI, USA), respectively, after initiation of large volume ventilation. Rats in the sham group only received anesthesia. All rats were sacrificed with an over dose of pentobarbital sodium for tissue analyses after MV.

### Tissue analyses

#### PaO_2_ to FiO_2_ ratio

Arterial blood gases were analyzed using the Bayer Rapidlab 348 analyser (Bayer Diognostics, Germany) to calculate the ratio of PaO_2_ to FiO_2_. Arterial blood gas analyses were performed at baseline and after MV.

#### Serum and bronchoalveolar lavage fluid

Peripheral blood was collected and centrifuged at 1500 × g for 10 min. Cytokine levels were analyzed in serum.

Bronchoalveolar lavage fluid (BALF) was collected by injecting and withdrawing five aliquots of 50 ml 4 °C saline into the left lung. BALF was centrifuged (1000 × g for 15 min) and the supernatant was collected. Protein levels in BALF were detected with the Bradford method. Macrophages and neutrophils in BALF were counted using a cell counter chamber.

Prostaglandin E2, tumor necrosis factor-α (TNF-α) and interleukin (IL)-1β levels in BALF and serum, and neutrophil elastase levels in BALF, were evaluated with enzyme linked immunosorbent assay kits (Wuhan Boster Bio-Engineering Limited Company, Wuhan, Hubei, China), according to the manufacturers’ instructions, at baseline and after MV.

#### Pulmonary alveolocapillary permeability

Pulmonary alveolocapillary permeability was measured by sampling a portion of the right lung from sacrificed rats. The wet weight of right lung tissue was recorded immediately, and the dry weight was recorded after drying for 48 h at 60 °C. The ratio of wet to dry weight (W/D) was calculated to estimate the effect of parecoxib on pulmonary capillary permeability.

#### Oxidative stress reaction in VILI

To investigate the effect of parecoxib on the oxidative stress reaction, malondialdehyde (MDA) levels in BALF and myeloperoxidase (MPO) activity in lung tissue were detected with MDA and MPO assay kits (Nanjing Jiancheng Corp., Nanjing, Jiangsu, China).

#### Lung tissue histopathologic analysis

A portion of the right lung from sacrificed rats was fixed with 10% formalin and embedded in paraffin. Lung sections were stained with hematoxylin and eosin. Two pathologists independently evaluated lung injury under light microscopy based on an assessment of alveolar congestion, edema, neutrophil infiltration, hemorrhage, thickness and integrity of the alveoli, and formation of a hyaline membrane.

#### TUNEL staining of lung sections

Apoptosis in lung tissue was investigated with terminal deoxynucleotidyl transferase-mediated biotinylated deoxyuridine triphosphate nick end labeling (TUNEL) staining using the Apoptosis Assay kit (Roche Diagnostics GmbH, Science, Mannheim, German). Sections of right lung tissue from sacrificed rats were digested with proteinase K, rinsed with phosphate buffered solution, and incubated in TUNEL reaction mixture for 60 min at 37 °C. After quenching the endogenous peroxidase activity with hydrogen peroxide, sections were placed in extra-avidin peroxidase and diaminobenzidine solution. Sections were counterstained with Mayer-hematoxylin and dehydrated. Brown-stained nuclei were considered positive for apoptosis. Independently, two pathologists calculated the apoptosis index.

### Western blot analysis

A portion of right lung tissue was homogenized and the protein was extracted. Total protein concentration was measured with the Bradford method. Aliquots of protein homogenate were separated on polyacrylamide gels and protein was transferred onto polyvinylidene fluoride membranes. Polyvinylidene fluoride membranes were blocked with 5% milk, immersed in primary antibodies against COX-2 (ab15191, Abcam Biotechnology, Cambridge, United Kingdom), B cell lymphoma/leukemia-2 (Bcl-2), BcL-2-associated X protein (Bax), and incubated with horseradish peroxidase-linked secondary antibodies (Santa Cruz Biotechnology, Santa Cruz, California, United States of America). Bands were visualized using enhanced chemiluminescence.

#### Survival analysis

Eight rats in each group were allowed to recover spontaneous breath after large volume ventilation. All the rats received lidocaine infiltrated locally for pain relief. Survival for 24 h after VILI was assessed. Moribund animals were defined as bradycardic, with a heart rate < 40 beats per minute, severely lethargic, and unresponsive to painful stimulation.

### Statistical analysis

All data were analyzed with SPSS software version 11.0 (SPSS, Chicago, Illinois, USA). Sample size was calculated based on a previous study, which showed that a sample size of 8 rats in each group was required to detect a 20% increase in PaO_2_/FiO_2_ with an alpha of 0.05 and a beta of 20%. Normally distributed data are presented as means ± standard deviation and were analyzed using the unpaired *t*-test. Non-normally distributed data were analyzed with the nonparametric Friedman test. A *P* value of <0.05 was considered statistically significant [18].

## Results

### Parecoxib improved gas exchange and pulmonary capillary permeability in VILI

**Fig. 1:**
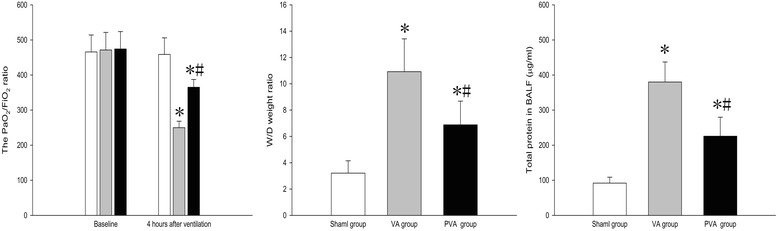
PaO_2_/FiO_2_, ratio of W/D weight in lung tissue, and protein concentration in BALF. Parecoxib significantly upregulated PaO_2_/FiO_2_ and decreased the W/D weight ratio in lung tissue and the total protein level in BALF after MV. *P < 0.05 compared with sham group; #*P* < 0.05 compared with VA group (, sham group; , VA group; , PVA group) (*n* = 8 in each group)

Compared to the S group, the PaO_2_/FiO_2_ ratio was decreased after 4 h of MV in the VA and PVA groups (both *P* < 0.001) (Fig. 1a), and the ratio of W/D weight and protein concentration in BALF were dramatically increased (both *P* < 0.05) (Fig. 1b and c).

### Parecoxib inhibited the oxidative stress response in VILI

**Fig. 2:**
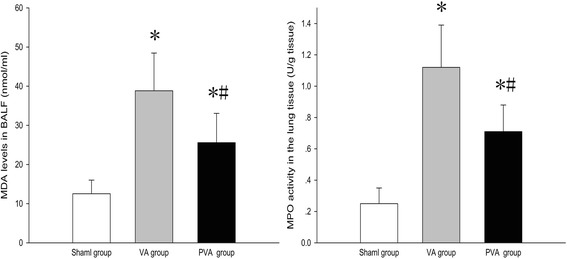
MDA level in BALF and MPO activity in lung tissue. Parecoxib significantly reduced MDA level in BALF and MPO activity in lung tissue after MV. *P < 0.05 compared with sham group; #*P* < 0.05 compared with VA group (*n* = 8 in each group)

Compared to the S group, MDA levels and MPO activity in lung tissue were increased in rats in the VA and PVA groups (both *P* < 0.05) (Fig. 2a and b).

### Parecoxib inhibited inflammation in VILI

**Fig. 3:**
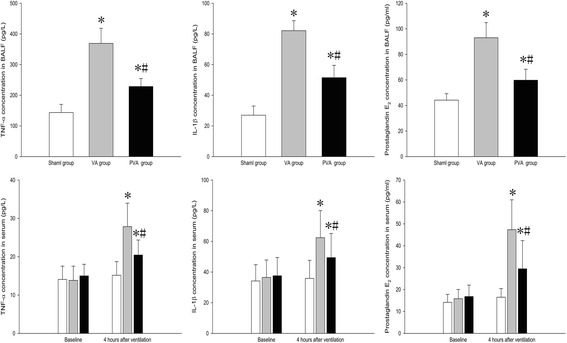
TNF-α, IL-1β, IL-8, and prostaglandin E_2_ levels in serum and BALF a. TNF-α, IL-1β, and prostaglandin E_2_ levels in serum and BALF were significantly decreased in rats treated with parecoxib (PVA) compared to saline-treated rats (VA group). *P < 0.05 compared with sham group; #*P* < 0.05 compared with VA group. (, sham group; , VA group; , PVA group) (*n* = 8 in each group)

Compared to the S group, TNF-α, IL-1β, and prostaglandin E_2_ levels in serum and BALF were increased in the VA and PVA groups (*P* < 0.05). Compared to the VA group, TNF-α, IL-1β, and prostaglandin E_2_ levels in serum (Fig. 3a, b and c) and BALF (Fig. 3d, e and f) were decreased in the PVA group (*P* < 0.05).

**Fig. 4:**
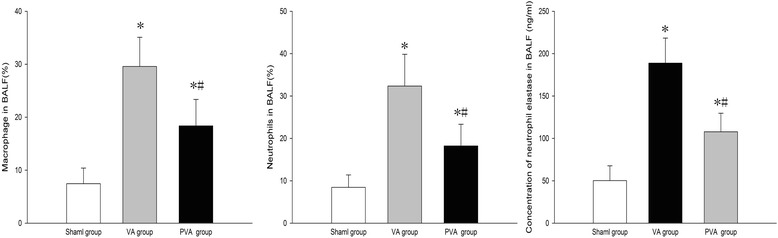
Macrophage and neutrophils, and neutrophil elastase levels in BALF. Parecoxib decreased the infiltration of macrophages and neutrophils induced by MV, and inhibited the release of elastase. *P < 0.05 compared with sham group; #*P* < 0.05 compared VA group (*n* = 8 in each group)

Compared to the S group, the macrophage ratio, neutrophil ratio and neutrophil elatase levels in BALF were increased in the VA and PVA groups (*P* < 0.05). Compared to the VA group, the macrophage ratio (Fig. 4a), neutrophil ratio (Fig. 4b), and neutrophil elatase levels (Fig. 4c) in BALF were decreased in the PVA group (*P* < 0.05).

**Fig. 5:**
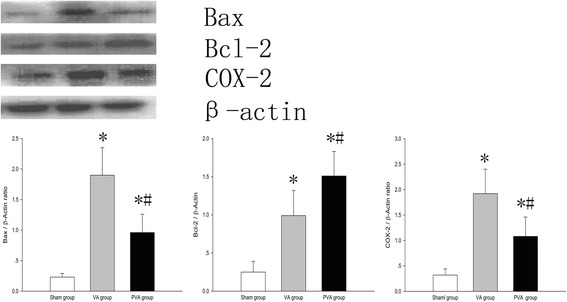
COX-2 protein levels in lung tissues. COX-2 protein level in lung tissues was significantly decreased in rats treated with parecoxib (PVA) compared to saline-treated rats (VA group). *P < 0.05 compared with sham group; #*P* < 0.05 compared with VA group. (, sham group; , VA group; , PVA group) (*n* = 8 in each group)

Compared to the S group, COX-2 and Bax protein levels in lung tissue were increased in the VA and PVA groups (*P* < 0.05) (Fig. 5a). Compared to the VA group, Bax (Fig. 5b) and COX-2 (Fig. 5d) levels in lung tissue were decreased, but Bcl-2 protein level (Fig. 5c) was increased, in rats in the PVA group (*P* < 0.05).

### Parecoxib attenuated lung injury in VILI

**Fig. 6:**
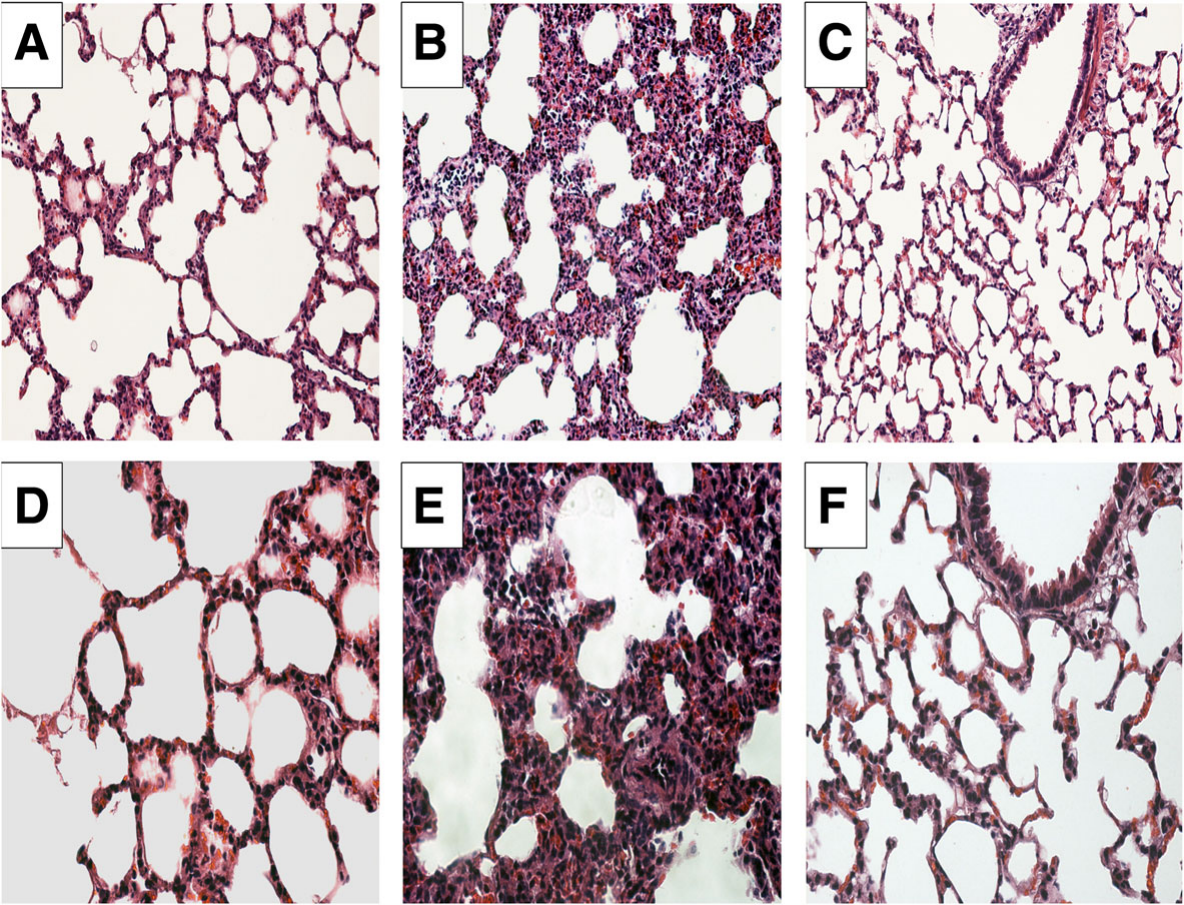
Histopathological analysis of lung tissue. **a**, **d** Sham group. **b**, **e** VA group. **c**, **f** PVA group. **a**–**c** × 200. **d**–**f** × 400. Lung tissue in the VA group showed thickened alveolar walls, edema, decreased alveolar space, and obvious inflammatory cell infiltration and hemorrhage. Parecoxib significantly decreased the degree of MV-induced histopathological injury (*n* = 8 in each group)

Light microscopic evaluation of the right lung revealed normal histology in rats in the S group (Fig. 6a, d). Compared to the S group, severe pathophysiological changes were seen in the right lungs of rats in the VA (Fig. 6b, e) and PVA groups (Fig. 6c, f), including collapse of alveoli, thickening of alveoli walls, severe diffused edema of alveoli and the interstitial space, formation of a hyaline membrane, infiltration of red blood cells, hemorrhage, and substantial infiltration of neutrophils and macrophages in lung parenchyma. In the PVA group, this pathophysiology was obviously attenuated.

### Parecoxib reduced apoptosis in VILI

**Fig. 7:**
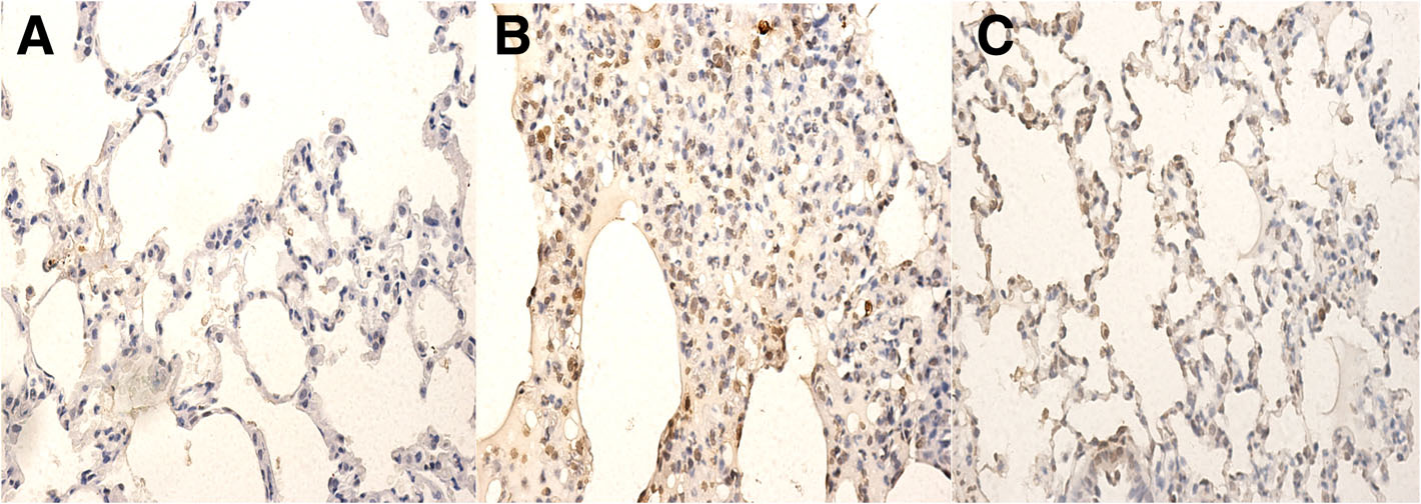
Apoptosis in lung tissues. Representative images of TUNEL staining of lung tissues in S group **a**, VA group **b** and PVA group **c. a**-**c**: ×400. The apoptosis index in lung tissues was significantly decreased in rats treated with parecoxib (PVA) compared to saline-treated rats (VA group). *P <0.05, vs. S group; #*P* <0.05, vs. VA group (*n* = 8 in each group)

Apoptotic epithelial and endothelial cells in lung tissue were observed in the VA and PVA groups, but not the S group (Fig. 7a). Compared to the VA group, apoptotic cells were dramatically decreased in the PVA group (Fig. 7b, c) (*P* < 0.05).

### Parecoxib improved survival following VILI

**Fig. 8:**
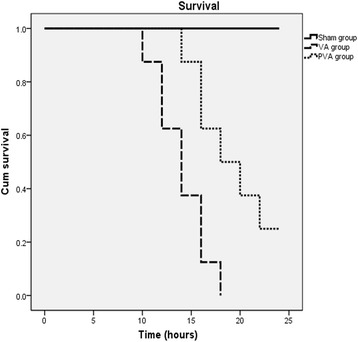
Survival time. Survival time was significantly prolonged by parecoxib compared to the VA group (*P* <0.001) (*n* = 8 in each group)

Compared to the VA group, survival time in the PVA group was significantly prolonged (*P* < 0.001) (Fig. 8).

## Discussion

The results of this study show that parecoxib improved gas exchange function and ameliorated VILI in a rat ARDS model. Parecoxib reduced histopathogical changes of VILI and apoptosis, as well as local and systematic inflammation compared to saline-treated mechanically ventilated rats. More important, parecoxib improved long term outcomes and increased survival after VILI in ARDS.

In VILI, mechanical overstretching of epithelial and endothelial cells can activate NF-κB and promote the release of chemoattractant and proinflammatory factors, which activate pulmonary macrophages and recruit neutrophils [19, 20]. Activated macrophages and neutrophils secrete vast amounts of pro-inflammatory cytokines and elastase leading to pulmonary endothelial injury, hypoxemia, and lung edema. Patients in the intensive care unit with ARDS usually require MV, and these patients are particularly susceptible to VILI because of lung inflammation. COX-2 is widely expressed in different inflammatory cells, pulmonary endothelium and epithelium, and has been associated with the pathophysiology of VILI and ARDS [11, 14, 17, 21, 22]. Induction of COX-2 results in an increase in prostaglandin E_2_ and prostacyclin levels, which play important roles in inflammation in the lung. Several studies show that inhibition or disruption of the COX-2 gene attenuates VILI and ARDS [7, 23, 24]. A recent report demonstrated that inhibition of COX-2 significantly decreased COX activity and attenuated VILI [17]. Although interesting, the clinical relevance of these findings is limited. A previous study from our laboratory indicates that inhibition of prostaglandin E_2_ and the COX-2 pathway by parecoxib attenuates acute lung injury induced by meconium [13]. Parecoxib is widely applied in clinical settings; therefore, in current study, we observed the effect of parecoxib on VILI in ARDS, in which prior lung injury or ARDS was induced with an intravenous injection of endotoxin.

We analyzed the effect of parecoxib on pulmonary gas exchange function, pulmonary capillary permeability, and histology in rats with ARDS after 4 h of MV. An increase in lung permeability induced by VILI can result in severe hypoxemia and lung edema. In this study, parecoxib increased the PaO_2_/FiO_2_ ratio, decreased the ratio of lung W/D weight and the protein content in BALF, and attenuated histopathogical changes after MV in rats. The findings suggested that parecoxib can reduce lung injury, prevent deterioration of alveolocapillary membrane function, and decrease lung edema in a rat model of ARDS and VILI.

We speculate that our findings are attributed to the anti-oxidative and anti-inflammatory properties of parecoxib. Results from previous studies have indicated that an oxidative stress response plays an important role in VILI [25–27]. During VILI, activated neutrophils release proinflammatory cytokines and reactive oxygen species, which directly damage pulmonary endothelial and epithelial cell membranes [28, 29]. In the present study, parecoxib significantly reduced MDA levels in BALF. MDA is the end product of lipid peroxidation, and MDA concentrations in serum are directly proportional to the severity of tissue damage caused by reactive oxygen species [30, 31]. In addition, parecoxib significantly decreased MPO activity. MPO plays a pivotal role in the oxidative stress response. MPO mediates peroxidation of chloride ions to form hypochlorous acid, which can directly damage lung epithelial and endothelial cells. MPO is also a significant source of tyrosine nitration, which can evoke protein conformational changes and damage to cell membranes [32, 33]. Evidence suggests that MPO promotes oxidation and nitrification during ischemia and reperfusion [34].

Inflammation plays a key role in VILI [35]. MV with high pressure or a large tidal volume can increase pulmonary hyperpermeability and contribute to pulmonary and systemic inflammation [36]. Furthermore, reactive oxygen species generated during VILI cause direct cellular damage, lead to severe local inflammation, and result in rapid transcription of chemokines and pro-inflammatory cytokines [37]. In the current study, parecoxib decreased levels of TNF-α and IL-1β in BALF and serum, the level of neutrophil elastase in BALF, and histopathogical injury in the lung tissue after MV in rats. These results suggest that parecoxib inhibited local and systemic inflammation. Anti-inflammatory effects of parecoxib may be medicated through the inhibition of prostaglandin E_2_. Prostaglandin E_2_, which is released from epithelial cells, can modulate immune and inflammatory responses [38, 39]. In the present study, prostaglandin E_2_ levels in BALF and serum and COX-2 levels in lung were significantly decreased by parecoxib after MV. We speculate that parecoxib ameliorated VILI through inhibition of COX-2, which attenuated prostaglandin E_2_ production. The anti-inflammatory effect of parecoxib may also result from an inhibition of neutrophil motility. In the present study, parecoxib significantly decreased the number of neutrophils in BALF after MV. Furthermore, parecoxib inhibited MPO activity, which is abundantly expressed in neutrophils, and usually correlates well with neutrophil count [40].

Both inflammation and the oxidative stress response can result in apoptosis, and apoptosis of endothelia and epithelia cells play a pivotal role in VILI [25, 41]. In the current study, we found that apoptosis was significantly inhibited by parecoxib. This result is in accordance with findings from previous studies [42, 43]. We detected the levels of Bax and Bcl-2 proteins to explore the role of parecoxib in apoptosis. Bax is an important pro-apoptotic protein [44]. In contrast, Bcl-2 can inhibit Bax activation and attenuate apoptosis. The Bax/Bcl-2 ratio is essential for controlling apoptosis. In the current study, parceoxib decreased the level of Bax protein in lung tissue, but increased the level of Bcl-2 protein. These results suggest that parecoxib decreased apoptosis by moderating the Bax/ Bcl-2 ratio.

## Conclusions

The results of this present study indicate that parecoxib can improve gas exchange function, decrease edema, reduce local and systemic inflammation, and ameliorate histological injury and apoptosis induced by VILI in an ARDS rat model. These findings suggest parecoxib may be a novel therapeutic option for ARDS patients undergoing MV.

## Abbreviations

ARDS: Acute respiratory distress syndrome
BALF: Bronchoalveolar lavage fluid
COX-2: Cyclooxygenase-2
IL: Interleukin
MPO: Myeloperoxidase
MV: Mechanical ventilation
PaO_2_/FiO_2_: Arterial oxygen partial pressure/fractional inspired oxygen
TNF: Tumor necrosis factor
VILI: Ventilator induced lung injury
W/D: Wet/dry weight
